# *Lactobacillus fermentum* CECT5716 Alleviates the Inflammatory Response in Asthma by Regulating TLR2/TLR4 Expression

**DOI:** 10.3389/fnut.2022.931427

**Published:** 2022-07-14

**Authors:** Weifang Wang, Yunfeng Li, Guojing Han, Aimin Li, Xiaomei Kong

**Affiliations:** ^1^Department of Respiratory and Critical Care Medicine, The Eighth Medical Center of the PLA General Hospital, Beijing, China; ^2^Hebei Key Laboratory of Chinese Medicine Research on Cardio-Cerebrovascular Disease, Hebei University of Chinese Medicine, Shijiazhuang, China; ^3^NHC Key Laboratory of Pneumoconiosis, Shanxi Key Laboratory of Respiratory Diseases, Department of Pulmonary and Critical Care Medicine, The First Hospital of Shanxi Medical University, Taiyuan, China

**Keywords:** asthma, *Lactobacillus fermentum* CECT5716, gut microbiota, TLR2, TLR4, lung

## Abstract

**Background:**

Asthma is a chronic disease, which is harmful to the health of the body and the quality of life. Supplementation of *Lactobacillus* can affect the immune environment of the lungs through the gut-lung axis. This study aimed to explore the potential regulatory targets of *Lactobacillus* to relieve inflammation in asthma and determine a new approach for improving asthma.

**Methods:**

A mouse ovalbumin (OVA)-induced model was constructed. OVA mice were supplemented with *Lactobacillus fermentum* CECT5716 by gavage. The gut microbiota composition of normal and OVA mice was analyzed using 16S ribosomal DNA identification. BALF, serum, lung tissues, and duodenal tissues were collected. Wright’s staining was performed to determine the cell content of the alveolar lavage fluid. Hematoxylin-eosin staining, Masson staining, and periodic acid-Schiff staining were performed to observe the improvement in the lungs of OVA mice supplemented with *Lactobacillus*. Immunofluorescence was performed to measure the severity of the intestinal barrier leakage. Enzyme-linked immunosorbent assay was carried out to determine the expression levels of inflammatory cell factors, while quantitative reverse transcription-polymerase chain reaction and western blotting were performed to detect the levels of toll-like receptor 2 (TLR2)/TLR4 expression and cell adhesion factors.

**Results:**

Compared with Control mice, OVA mice exhibited malignant conditions, such as intestinal leakage and lung edema. After supplementation with *Lactobacillus*, the inflammatory cell content in the bronchoalveolar lavage fluid decreased, and the inflammatory response was alleviated. The level of TLR2/TLR4 expression was reduced. The inflammatory cell infiltration in the airway mucosa of OVA mice was improved, alveolar swelling was reduced and the basement membrane appeared thinner.

**Conclusion:**

The *Lactobacillus* inhibited the TLR2/TLR4 expression in OVA mice. Supplementation with *Lactobacillus* can alleviate the inflammatory response in OVA mice, inhibit pulmonary fibrosis, and treat asthma.

## Introduction

Asthma is a chronic bronchial inflammatory disease involving various cells and cytokines (1). The clinical manifestations are mainly wheezing, shortness of breath, chest tightness, cough and other symptoms, and are affected by environmental stimuli and genetic factors (2). The currently available treatment options alleviate the symptoms of asthma and other allergic diseases, but do not provide a complete cure. Most people with asthma tend to boost their immunity by taking specific microbial food supplements or probiotics that can potentially prevent or treat allergic diseases (3). It is widely recognized that commensal microorganisms are relevant for human and animal health, participating in several important biological functions, including nutrient digestion, vitamin synthesis, and pathogens inhibition (4, 5). Probiotics have anti-inflammatory effects and anti-cancer effects, induce beneficial bacterial proliferation, suppress harmful bacteria, improve intestinal health, reduce blood cholesterol levels, and suppress endogenous infections (6). Lactic acid bacteria are a probiotic that prevents the early development of allergic diseases in children and mouse asthma models (7). In many studies, the oral administration of probiotics such as *Lactobacillus rhamnosus* GG, *Lactobacillus gasseri, Lactobacillus fermentum* NWS29, *Lactobacillus casei* NWP08, *Lactobacillus rhamnosus* NWP13, and *Lactobacillus salivarius* PM-A0006 have shown significant benefits in mouse models of allergic asthma (8). In different experimental models, *Lactobacillus fermentum* CECT5716 has shown anti-inflammatory effects and capacity to modulate microbiota composition (9). It suggests a potential use of *Lactobacillus fermentum* CECT5716 in clinical practice. However, no association between *Lactobacillus fermentum* CECT5716 and asthma has been reported yet. This study was conducted to investigate the effects of *Lactobacillus fermentum* CECT5716 on asthma prevention using a murine model of asthma.

Well-documented examples of probiotic-effector molecules in *Lactobacillus* and *Bifidobacterium* strains include surface-localized molecules such as specific pili, S-layer proteins, extracellular polysaccharides, polypeptides; more broadly produced metabolites such as tryptophan-associated and histamine-associated metabolites; CpG-rich DNA; and various enzymes (such as lactase and bile salt hydrolases) (10). The gut microbiota is an important stimulator of the development and function of the immune system and plays an important role in regulating immune responses. The composition and metabolites of the microbiota of allergic individuals are different from those of healthy individuals (11). In addition, asthma involves disturbances in the gut microbiota and is associated with metabolic dysfunction. Manipulation of the gut microbiota using oral probiotics or high-fiber dietary supplements that increase the synthesis of short-chain fatty acids contributes to the affinitive local and distal mucosal immunity in mice (12). Therefore, modulating the gut microbiota using probiotics and through dietary changes is a reasonable treatment to prevent asthma and other allergic diseases (13). In this study, we focused on improving the gut microbiota using *Lactobacillus fermentum* CECT5716 as the direction of research.

Gut microbiota can help maintain the normal functioning of the human body through specific signaling pathways. Toll-like receptors (TLRs) expressed by antigen presenting cells (macrophages, dendritic cells) and other various cells (e.g., airway epithelial cells) are innate immune sensors, which recognize microbial pathogen-associated molecular patterns (bacteria, fungi, and virus structures) as well as endogenous danger molecules from host cells (14). It reported that TLR2 and TLR4 is a key protein in OVA-induced exacerbation of murine lung eosinophilia by Asian sand dust (15). Pharmacological and probiotic methods for specific toll-like receptor 2 (TLR2) signaling processes can be developed to treat colonic motility diseases related to the use of antibiotics or other factors (16). rs3804099 in TLR2 and rs4986791 in TLR4 are significantly associated with an increased risk of asthma. The polymorphism of TLRs plays an important role in the occurrence of asthma (17). The TLR2, TLR4, and MyD88 have important and complex roles in promoting the development of OVA-induced antibiotic-associated diarrhea. Therefore, the activation of TLR signaling may be required for developing and suppressing asthma induced by *Streptococcus pneumoniae* and establishing other potential immunomodulatory therapies (18). After *Streptococcus pneumoniae* infection, the accumulation of eosinophils in the bronchoalveolar lavage fluid (BALF) and blood, the release of Th2 cytokines from the mediastinal lymph nodes, spleen cells, and aryl hydrocarbon receptor (AHR) was observed (19). Previous studies have shown that TLR2/TLR4 can promote the expression of inflammatory factors in the Th17 cells, including interleukin (IL)-4, IL-5, and IL-17 (20). In induced sputum, asthma patients with higher total serum immunoglobulin (IgE) levels showed increased macrophage expression of TLR4. This result may be due to the relationship between innate immunity and the IgE-mediated adaptive immune response in asthma. The TLR4 is a potential therapeutic target (21). To study the relationship between the gut microbiota and the TLR2/TLR4 expression, we constructed an OVA mouse model to observe the pathological improvement.

Previous studies have shown that the gut microbiota may affect the signaling pathway to improve asthma. We found that the gut microbiota of asthmatic mice was disturbed. We explored whether the administration of *Lactobacillus fermentum* CECT5716 could reduce the TLR2/TLR4 expression, inhibit the production of inflammatory factors by inflammatory cells, decrease intestinal leakage and pneumonia, and treat asthma. These findings provide a good basis for the treatment of asthma.

## Materials and Methods

### Strain Preparation

*Lactobacillus fermentum* CECT5716 was provided from Hangzhou Hongsai Biotechnology Co., Ltd. Lactobacilli strains were grown in Mann–Rogosa–Sharpe Agar (MRS) (CM1153R, Thermo, China) at 37°C. After overnight growth, bacteria were transferred to MRS broth and cultured at 37°C until the stationary phase. Afterwards, the bacteria were pelleted (3000 × g for 10 min), washed twice with phosphate-buffered saline (PBS) and suspended in specific media for *in vivo* or *in vitro* assays.

### Experimental Design

Eight-week-old C57BL/6 mice weighing 22 ± 2 g were purchased from SiPeiFu Biotechnology Co., Ltd. During the experiment, the care and use of laboratory animals complied with the “Guiding Opinions on the Good Treatment of Laboratory Animals” issued by the Ministry of Science and Technology in 2006. The asthmatic mice were sensitized by intraperitoneally injecting 0.2 mL of PBS containing 20 μg OVA (O1641, Sigma-Aldrich, Shanghai) and 2 mg alum on the 1st and 5th days and then exposing them to 1% OVA using a nebulizer on the 12th and 13th days of atomization (w/v) for 40 min. The mice in the control group were injected with 200 uL PBS at the same time. After 24 h, BALF serum, lung tissues, and duodenal tissues were collected for subsequent testing. Each of the following groups consisted of five mice: Control group (normal mice injected with 200 uL PBS), OVA group (asthma model mice injected with 200 uL PBS), and OVA + probiotics group. Twenty-four hours after the second sensitization, 10^10^ CFU (200 uL of PBS) of *Lactobacillus fermentum* CECT5716 was administered to the OVA group by gavage once a day for 4 weeks (22). For euthanasia, mice were anesthetized with isoflurane (4%) and then inoculated with an intraperitoneal injection of pentobarbital (0.5 mL per mouse). Mice were sacrificed on day 56. Blood for total IgE, bronchoalveolar lavage fluid (BALF), lung tissues and duodenal tissues for cytokine analysis were collected and subsequently analyzed as previously described (23).

### Hematoxylin-Eosin Staining

Lung tissues from different groups of mice were used for Hematoxylin-eosin staining. The lung tissues were fixed in 4% paraformaldehyde for 24 h and embedded in paraffin. Then, the tissues were cut into slices. The slices were then placed in xylene for 15 min three times; immersed in 100, 100, 95, 85, and 75% ethanol for 5 min; and soaked in distilled water for 5 min. The sections were stained with hematoxylin (H301933, Aladdin, China) for 3 min and washed with distilled water. Next, the sections were stained with eosin (E196384, Aladdin, China) for 5 s, dehydrated with gradient alcohol (95–100%) for 5 min, and then removed and placed twice in xylene for 10 min. Finally, the sections were sealed with a neutral gum and observed under a microscope (BA210T, Motic, China).

### Masson Staining

The whole tissue was covered with an appropriate amount of nuclear staining solution and stained for 3–5 min. The staining solution was rinsed with tap water. The slices (AY89-2, Yuantai, China) were soaked in distilled water. The sections were soaked in weak alkaline solvents such as PBS or ammonia for 5–10 min until the nuclei appeared blue. The slices were separated with a color separation solution for 30 s and rinsed with absolute alcohol until they appeared transparent, blow-dried, and sealed.

### Quantitative Reverse Transcription-Polymerase Chain Reaction

Subsequently, qRT-PCR was performed on a fluorescence qPCR instrument (QuantStudio1, Thermo, United States) using an UltraSYBR Mixture (CW2601, CWBIO, China). The following reaction conditions were used: pre-denaturation at 95°C for 10 min, 40 cycles of denaturation at 94°C for 15 s, and annealing at 60°C for 30s. β-actin was used as an internal reference primer; the primer sequences are shown in Table 1. The relative quantitative method (2^–△^^△^ *^Ct^* method) was used to calculate the relative transcription level of the target gene: △△Ct = △ experimental group – △ control group, △Ct = Ct (target gene) – Ct (β-actin).

**TABLE 1:** Primer sequences.

Gene	Sequences (5′-3′)
TLR4	F: AGACACTTTATTCAGAGCCGTTG
	R: AAGGCGATACAATTCCACC
TLR2	F: CTCTTCAGCAAACGCTGTTCT
	R: GGCGTCTCCCTCTATTGTATTG
β-actin	F: ACATCCGTAAAGACCTCTATGCC
	R: TACTCCTGCTTGCTGATCCAC

### Western Blot

The protein was separated by sodium dodecyl sulphate–polyacrylamide gel electrophoresis using 10% gel and transferred to the NC membrane by electrotransfer. The membrane was blocked with 5% skimmed milk for 2 h at room temperature to allow it to bind non-specifically and incubated with the primary antibody overnight at 4°C. The primary antibodies used were rabbit anti-TLR2 (1:2000, 66645-1-Ig, Proteintech), rabbit anti-TLR4 (1:500, 19811-1-AP, Proteintech), rabbit anti-E-cad (1:5000, 20874-1-AP, Proteintech), rabbit anti-Occludin (1:3000, 27260-1-AP, Proteintech), rabbit anti-ZO-1 (1:8000, 21773-1-AP, Proteintech), rabbit anti-Claudin-2 (1:800, ab204049, Abcam), and rabbit anti-β-actin (1:5000, 60008-1-Ig, Proteintech). The membrane was washed three times with TBST for 10 min. Next, the HRP-labeled HRP goat anti-mouse IgG (1:5000, SA00001-1, Proteintech) was incubated. The film was immersed in SuperECL Plus (K-12045-D50, Advansta, United States) to observe for luminescence. β-actin was used as the internal reference. The target band was analyzed using ImageJ software.

### 16S Ribosomal RNA Identification

We sent the collection of fecal samples to the Apexbio (Shanghai, China) for 16S rDNA analysis. The Qiime 2 software was used to calculate the alpha diversity index of each sample, draw the rank-abundance curve based on OTU, and create the dilution curve based on alpha diversity. Meanwhile, the R software was used to draw the relative abundance histograms of species (R ggplot2 package), genus-level abundance heat maps (R reshape2/ggplot2 package), box plots of the differences in alpha diversity between groups based on the Wilcoxon test (between two groups) (R phyloseq package), PCA dimensionality reduction analysis map based on the Bray-Curtis distance (phyloseq/vegan package), sample clustering tree based on the Euclidean distance (R built-in function), and ANOSIM of bacterial abundance difference map (R DESeq2 package). In addition, web analysis tools were utilized to create the Venn diagram and perform the Lefse analysis, picrust2 function prediction, and Krona species composition analysis.

### Immunofluorescence

All the duodenum tissues were fixed in 4% paraformaldehyde overnight and embedded in paraffin with standard techniques. Then, the tissues were cut into slices. The slices were placed in xylene for 20 min; immersed in 100, 95, 85, and 75% ethanol sequentially for 5 min at each level; and then immersed in 0.01 M citrate buffer (pH 6.0). After continuously boiling the slices for 20 min, they were removed after cooling for 20 min and cooled to room temperature. Appropriately diluted primary antibodies against E-cadherin (1:50, ab15098, Abcam), Occludin (1:50, 20874-1-AP, PTG), ZO-1 (1:50, 27260-1-AP, PTG), and Claudin-2 (1:50, 21779-1-AP, PTG) were added to the slices. Approximately 50–100 uL of anti-rabbit-IgG-labeled fluorescent antibody were also added on the slices and incubated at 37°C for 90 min. A DAPI working solution was used to stain the nucleus for 10 min at 37°C. Finally, the results were observed under a fluorescence microscope.

### Wright’s Staining

A total of 50 μL of Ray Dahl staining reagent (D010-1-2, Jiancheng, China) was added to 2 μL of suspended cell smear; the tissue section was stained for 1 min. Then, 100 μL of differentiation solution was directly added to the smear. The glass slide was shaken gently until the dye was completely mixed with the sample, and then the sample was dyed for 5 min. The smear was placed twice on xylene for 10 min. The cytoplasm of monocytes appeared grayish blue, while that of lymphocytes appeared light blue.

### Enzyme-Linked Immunosorbent Assay

After blood collection, the chest was opened, the complete trachea, bronchus and lung tissue were isolated, the right main bronchus was ligated, and 2 mL of normal saline was injected into the left lung lobe through the left bronchus with a lavage device connected to a syringe, and slowly aspirated three times. Then suck it out with negative pressure and repeat twice. After centrifuging the lavage solution at 3,000 rpm × 10 min, 0.5 mL of the supernatant lavage solution was lyophilized. The IL-4 (CSB-E04634M), IL-5 (CSB-E04637M), IL-13 (CSB-E04602M), IL-12 (CSB-E04600M), IFN-γ (CSB-E04578M), and IgE (CSB-E07983m) kits (Wuhan Huamei Biological Engineering Co., Ltd.) were used for the experiments (24). We removed a standard from the kit and centrifuged it at 6,000-10,000 rpm for 30 s. The concentrated washing solution was diluted 1:25 with deionized water. Biotin-labeled antibody solution was diluted 1:100 times with biotin-labeled antibody diluent. Standard and sample wells were separately set and tested; then, 100 μL of standard or sample was added to each well, the sample was shaken gently in order to mix, the plate was covered with stickers, and the sample was incubated at 37°C for 2 h. Then, 100 μL of biotin-labeled antibody working solution was added to each well. Each hole was covered with a new board. The kit was incubated at 37°C for 1 h. Approximately 100 μL of horseradish peroxidase-labeled avidin working solution was added to each well. Next, 90% substrate solution was added to each well, and then the sample was incubated at 37°C for 15–30 min. Finally, 50 μL of stop solution was added to each hole in sequence. Five minutes after terminating the reaction, the optical density of each pore was measured using a microplate at 450 nm.

### Periodic Acid-Schiff Staining

First, the slices were immersed twice in xylene for 10 min; placed in 100, 95, 85, and 75% ethanol for 5 min; and then soaked in distilled water for 5 min. Next, 50 ul of periodate was added to the slices and allowed to stand for 10 min. The slices were washed with tap water for 10 min, stained with Schiff’s solution (S378685, Aladdin, China) for 10 minutes, and then stained with hematoxylin for 5–10 min. Finally, the sections were observed under a microscope.

### Statistical Analysis

All data were analyzed using GraphPad Prism 8.0 software (GraphPad Software, San Diego, California, United States). The measurement data were expressed as mean ± standard deviation (SD). The two sets of data conforming to the normal distribution were used unpaired T test. The multiple sets of data conforming to the normal distribution adopt one-way analysis of variance, and then perform Tukey’s *post hoc* test. A P value of < 0.05 was considered significant.

## Results

### The Expression of TLR2/TLR4 Protein Was Activated in the Ovalbumin Group

**FIGURE 1:**
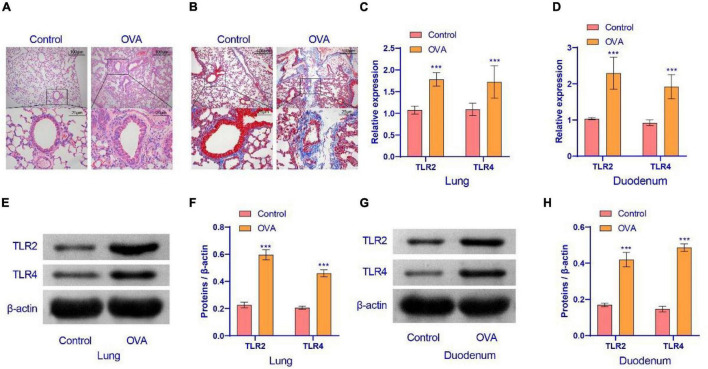
Airway inflammation and TLR2/TLR4 expression in the lung of asthma mice. **(A)** HE staining were performed on the lung tissues of mice. **(B)** Masson staining was performed on the lung tissues of mice. **(C,D)** qRT-PCR was used to detect the expression of mRNA. The level of TLR2/TLR4 mRNA was suppressed in the OVA group. **(E–H)** Western blot was used to measure the expression of proteins. The expression of TLR2/TLR4 protein was raised in the OVA group. up: × 100, scale bar = 100 μm; down: × 400, scale bar = 25 μm. ***Compared with the control group, *P* < 0.001. Unpaired *t*-test was utilized for the comparison between two groups. *n* = 5 for each group.

We constructed an OVA model to assess the pathological conditions of mice with asthma. In order to observe whether the OVA model was successful, HE staining and Masson staining were performed on the lung tissues of mice. The results are displayed in Figures 1A,B. OVA group compared with the control group, lung tissue had increased inflammatory cell infiltration. The base membrane was thickened. Deposition of collagen was seen not only in the basement membrane of bronchial epithelia and blood vessels, but also in lung parenchyma. These findings showed that the OVA mice were successfully established. Compared with the control group, TLR2/TLR4 expression in the lung and duodenum tissues of OVA-induced murine asthma model was increased (Figures 1C–H). In summary, the TLR2/TLR4 expression was activated in the OVA mice.

### Asthma Led to Gut Dysmicrobiota

**FIGURE 2:**
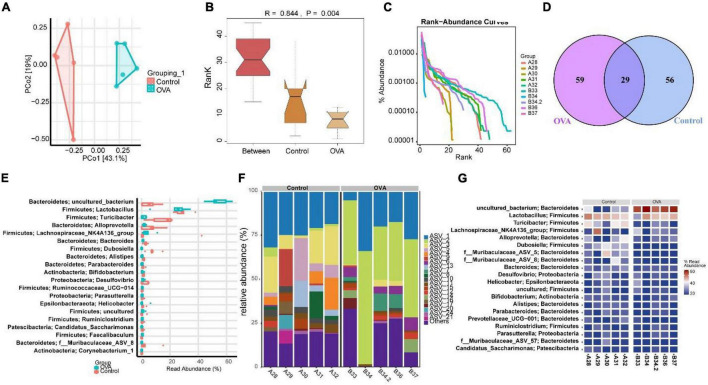
Asthma could cause gut dysmicrobiota. **(A)** PCA was performed to analyze the similarity of samples. **(B)** ANOSIM was used to analyze the difference between two samples. **(C)** Rank abundance. **(D)** OUT Venn diagram. **(E)** Box diagram of phylum. **(F)** Phylum distribution histogram. **(G)** Heat map of the phylum distribution.

The above results indicated that the TLR2/TLR4 expression in the duodenum interfered with the pathology of asthma. Therefore, we explored whether asthma affected the gut microbiota of mice. Principal component analysis (PCA) showed that the microbial community structure between the control and OVA groups was less similar (Figure 2A). The analysis of similarities (ANOSIM) obtained an R value of 0.844, showing that the between-group variance was larger than the within-group variance (Figure 2B). The rank-abundance curve showed that the OVA group curve had a larger range on the horizontal axis and a higher species richness (Figure 2C). The OUT Venn diagram showed that the control group had 56 unique OTU numbers, while the OVA group had 59 OTUs (Figure 2D). The results suggested significant differences in *Bacteroidetes* and *Firmicutes* between the control and OVA groups (Figure 2E). The heat map and the abundance bar graph showed substantial differences in the phylum composition between the control and OVA groups (Figures 2F,G). These results suggest that the gut microbiota of asthmatic mice is imbalanced.

### *Lactobacillus fermentum* CECT5716 Supplementation Could Reduce the Intestinal Barrier Leakage

**FIGURE 3:**
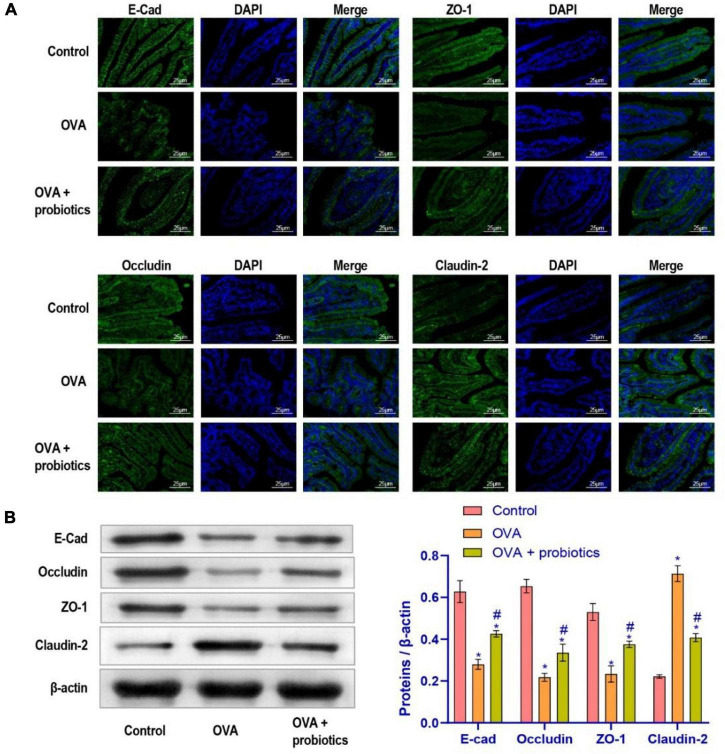
The leakage of intestinal barrier was alleviated after supplementation of Lactobacillus fermentum CECT5716. **(A)** Immunofluorescence imaging of E-cad, ZO-1, Occludin, and Claudin-2 (× 400, scale bar = 25 μm). **(B)** Western blot was used to measure the expression of proteins. Protein expression levels of E-cad, ZO-1, Occludin, and Claudin-2 in each group. *Compared with the control group, *P* < 0.05. ^#^Compared with the OVA group, *P* < 0.05. One-way ANOVA was used among multiple groups. *n* = 5 for each group.

The above experiments showed that asthma could cause gut dysmicrobiota and that the TLR2/TLR4 expression were disturbed. We examined whether *Lactobacillus* supplementation could improve the intestinal barrier leakage caused by disruption of the gut microbiota. Therefore, *Lactobacillus fermentum* CECT5716 was administered to evaluate its effect in the duodenal function of OVA mice. Immunofluorescence was performed to evaluate the expression levels of E-cadherin, ZO-1, Occludin, and Claudin-2 in the duodenum. Results showed that the duodenum of mice supplemented with was repaired (Figure 3A). Then, we examined the expression levels of adhesion link proteins. Compared with the OVA group, the E-cad, ZO-1, and Occludin protein expression levels in the OVA + probiotics group increased, while and the Claudin-2 protein level significantly decreased (Figure 3B). In short, results indicated that *Lactobacillus* supplementation could reduce the intestinal barrier leakage.

### *Lactobacillus fermentum* CECT5716 Could Inhibit the Level of Pro-inflammatory Factors in BALF

**FIGURE 4:**
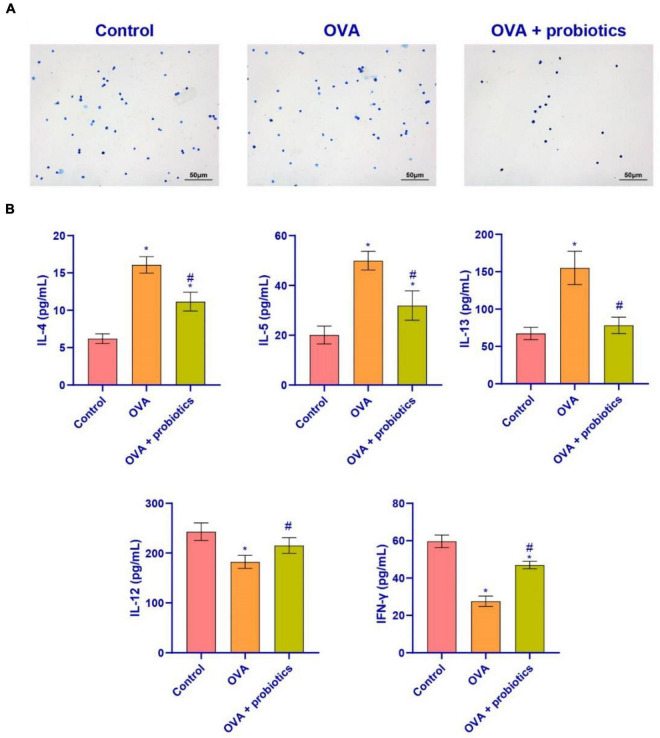
*Lactobacillus ferokay smentum* CECT5716 might inhibit the level of pro-inflammatory factors in BALF. **(A)** Wright’s staining (× 200, scale bar = 50 μm). **(B)** ELISA was used to measure the level of inflammatory factors. Expression levels of inflammatory factors (IL-4, IL-5, IL-13, IL-12, and IFN-γ). *Compared with the control group, *P* < 0.05. ^#^Compared with the OVA group, *P* < 0.05. One-way ANOVA was used among multiple groups. *n* = 5 for each group.

We explored further whether the lung function could be improved by supplementation with *Lactobacillus*. We extracted BALF from mice for the follow-up experiments. Wright’s staining was performed to detect the number of inflammatory cells in the BALF. The data suggested that the number of inflammatory cells decreased in the OVA + probiotics group. *Lactobacillus* supplementation inhibited the production of inflammatory cells (Figure 4A). ELISA was carried out to analyze the levels of pro-inflammatory and anti-inflammatory factors in Th2 cells found in the BALF. Compared with the OVA group, the levels of pro-inflammatory factors (IL-4, IL-5, and IL-13) in the OVA + probiotics group significantly decreased. Meanwhile, the levels of anti-inflammatory factors (IL-12 and interferon gamma [IFN-γ]) were significantly increased (Figure 4B). The above data showed that supplementation with *Lactobacillus* reduced the level of pro-inflammatory factors.

### *Lactobacillus fermentum* CECT5716 Could Inhibit the Expression of TLR2/TLR4

**FIGURE 5:**
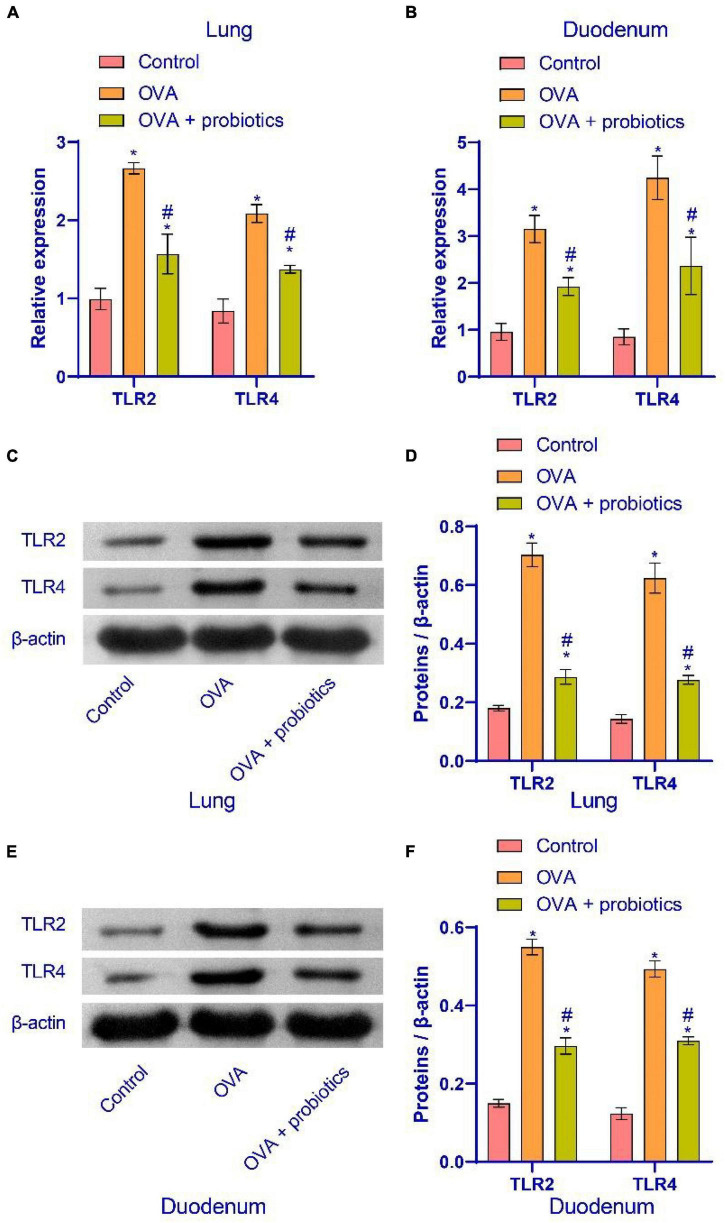
Supplementation with Lactobacillus fermentum CECT5716 could inhibit the TLR2/TLR4 expression. **(A,B)** qRT-PCR was used to detect the expression of mRNA. Lactobacillus supplementation could reduce the mRNA expression of TLR2 and TLR4. **(C–F)** Western blot was used to measure the expression of proteins. Lactobacillus supplementation could inhibit the protein expression of TLR2 and TLR4. *Compared with the control group, *P* < 0.05. ^#^Compared with the OVA group, *P* < 0.05. One-way ANOVA was used among multiple groups. *n* = 5 for each group.

The above experimental results indicated that *Lactobacillus* could alleviate asthmatic mice’ inflammation in the duodenum and lungs. We studied the effect of *Lactobacillus* on the TLR2/TLR4 expression in the duodenum and lungs of mice. qRT-PCR and western blotting were performed to detect the expression of TLR2/TLR4. In the OVA + probiotics group, the mRNA expression of TLR2 and TLR4 in the lungs of mice was downregulated. The protein expression of TLR2 and TLR4 reduced. The protein expression of TLR2 and TLR4 also decreased. Similar results were observed in the duodenum of this group (Figure 5). The results showed that *Lactobacillus* did not affect the expression of TLR2 and TLR4 in normal mice. This finding showed that the TLR2/TLR4 expression in OVA mice was decreased.

### *Lactobacillus fermentum* CECT5716 Could Reduce the Inflammatory Cell Infiltration

**FIGURE 6:**
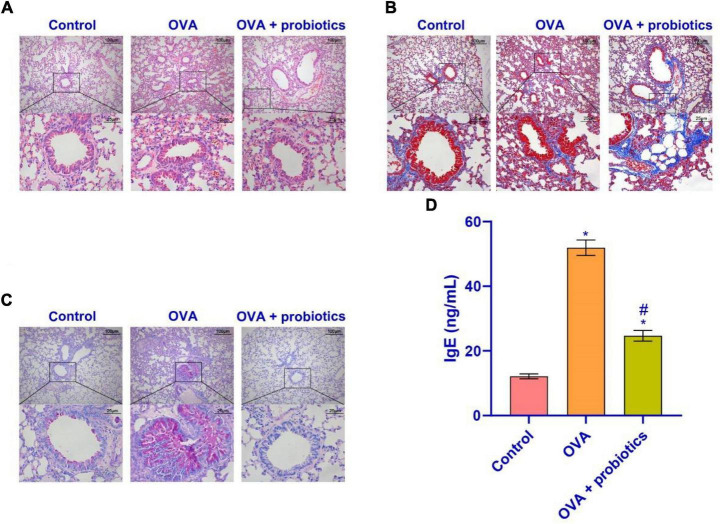
*Lactobacillus fermentum* CECT5716 could relieve the inflammatory cell infiltration. **(A)** HE staining was performed to determine the pathological changes in the lung tissue. **(B)** The degree of lung fibrosis was analyzed by Masson staining. **(C)** PAS staining was utilized to observe the proliferation of tracheal goblet cells in the lung tissues of each group. **(D)** Level of IgE in the serum. Up: × 100, scale bar = 100 μm; down: × 400, scale bar = 25 μm. *Compared with the control group, *P* < 0.05. ^#^Compared with the OVA group, *P* < 0.05. One-way ANOVA was used among multiple groups. *n* = 5 for each group.

The above results showed that supplementation with *Lactobacillus* could increase the TLR2/TLR4 expression. We observed the pathological conditions of the mice’s lungs. Hematoxylin-eosin (HE) staining, Masson staining, and PAS staining were used to observe the lungs of mice supplemented with *Lactobacillus*. Figures 6A–C shows that compared with the OVA group, the inflammatory cell infiltration in the airway mucosa in the OVA + probiotics group improved, the alveolar swelling was reduced, and basement membrane thinning. Finally, ELISA was used to detect the IgE content. Figure 6D shows that supplementation with *Lactobacillus* significantly inhibited the production of IgE. Overall, *Lactobacillus* supplementation can relieve lung inflammation.

## Discussion

This study aimed to determine whether *Lactobacillus fermentum* CECT5716 supplementation could improve the intestines and lungs of asthmatic mice. The TLR2/TLR4 expression may affect the secretion of inflammatory factors by the inflammatory cells, which regulate the target of asthma progression. *Lactobacillus* supplementation could regulate the gut microbiota composition by inhibiting the TLR2/TLR4 expression, thus relieving duodenal leakage and pulmonary inflammatory symptoms.

Asthma remains an important medical emergency, the most common cause of acute hospital admissions in children, and the most common chronic respiratory disease in adults (25). Anti-inflammatory and bronchodilator therapies are the main treatments for asthma (26). Chrysanthemum improved the OVA-induced allergic asthma by inhibiting the activation of pro-inflammatory cytokines and their upstream TLR/NF-κB/NLRP3 pathways (27). The oral administration of probiotics led to the suppression of serum OVA-specific IgE production through the TLR2 pathway. This enhances the production of IFN-γ in spleen cells that respond to OVA (28). Supplementation with *Lactobacillus fermentum* CECT5716 differentially modulates the immune response of IECs triggered by TLR4 activation through the regulation of the expression of negative TLR regulators (29). ILR mediates the secretion of cytokines by immune cells. In skin diseases, an increase in IL-4 and IL-13 levels is caused by abnormalities in TLR-mediated processes (30). In systemic inflammation, regulation of TLR could help improve the cell barrier function. The main reason for this is that the function of Occludin in cells is destroyed (31). The above research objects include active ingredients in plants and probiotics, which can improve asthma by affecting TLR2 or TLR4 pathway *in vivo*. Our choice of research objects are also probiotics. Firstly, we constructed OVA model mice. The results of HE and Masson staining showed that the infiltration of inflammatory cells in the lung tissue of OVA mice increased. The abnormal expression of TLR2/TLR4 in asthmatic mice is consistent with previous studies. The TLR2/TLR4 expression of OVA mice was activated and the intestinal flora was disordered. After that, we observed whether asthma was improved by supplementing OVA mice with *Lactobacillus fermentum* CECT5716. However, we did not elucidate the association between Lactobacillus fermentum and intestinal microbiota. This provided direction for the next step in our research program.

*Lactobacillus* is one of the most widely known probiotics (32, 33). Probiotics can reduce the autoimmune response of patients with rheumatoid arthritis by improving inflammation and disease activity (34). In addition, probiotics reduce the risk of islet autoimmunity in children with the highest genetic risk of type 1 diabetes (35). In ulcerative colitis, probiotics induce and maintain remission (36). However, little is known about the interactions between probiotics and host cells or how to manipulate these interactions to obtain a stronger regulatory response (37). In this sense, the sensor of innate immunity is the target of probiotics and plays an important role in the expression of TLRs (38). TLR4 was an important immune pattern recognition receptor, which can control the innate and adaptive immune responses, and played an important role in the initiation and regulation of airway inflammation (39). A few studies reported on the role of *Lactobacillus* in the treatment of asthma (8, 40). We conducted experiments to verify these findings and found that *Lactobacillus* species can provide basic beneficial effects, inhibit the inflammatory response in asthmatic mice, and inhibit the progression of lung fibrosis in mice. Studies have shown that asthma improves by repairing the gut barrier (41). Our results are consistent with those of previous studies. *Lactobacillus* supplementation in OVA mice up-regulated the expression of E-cad, ZO-1 and Occludin. It inhibited the expression of Claudin-2 protein to improve the intestinal barrier leakage caused by intestinal microecological disorder. TLR2 gene knockout in asthmatic mice can alleviate the airway inflammation, whose mechanism may be that the allergic airway inflammation of asthmatic mice is alleviated (42). Our results of pro-inflammatory factors (IL-4, IL-5, and IL-13) were significantly decreased in OVA mice supplemented with *Lactobacillus*, while the levels of anti-inflammatory factors (IL-12, IFN-γ) were significantly increased. *Lactobacillus fermentum* CECT5716 can inhibit the production of IgE in OVA mice. The TLR2/TLR4 expression in OVA mice was inhibited. However, we have not studied TLR2/TLR4 at the clinical level. We will conduct more in-depth research on TLR2/TLR4 in future research, and hope to make some clinical research progress.

In conclusion, this study found that in asthmatic mice, *Lactobacillus fermentum* CECT5716 could protect the intestines and lungs. We verified that the risk of intestinal leakage was curbed. The TLR2/TLR4 expression was inhibited, and the levels of inflammatory factors secreted by inflammatory cells in mice were reduced. The cavitation in the lungs was relieved. Results of our study clarified that *Lactobacillus fermentum* CECT5716 can inhibit the TLR2/TLR4 expression to relieve lung inflammation in asthmatic mice.

## Data Availability Statement

The datasets presented in this study can be found in online repositories. The names of the repository/repositories and accession number(s) can be found below: https://www.ncbi.nlm.nih.gov/, PRJNA739083.

## Ethics Statement

This study was approved by the Animal Experiment Ethics Committee of Hebei University of Chinese Medicine (No. DWLL2020077) and conducted in strict accordance with the national institutes of health guidelines for the care and use of experimental animals.

## Author Contributions

WW, YL, and GH had data collection and analysis, and manuscript preparation. AL and XK supervised the whole study, data analysis, and manuscript preparation. All authors contributed to the article and approved the submitted version.

## Conflict of Interest

The authors declare that the research was conducted in the absence of any commercial or financial relationships that could be construed as a potential conflict of interest.

## Publisher’s Note

All claims expressed in this article are solely those of the authors and do not necessarily represent those of their affiliated organizations, or those of the publisher, the editors and the reviewers. Any product that may be evaluated in this article, or claim that may be made by its manufacturer, is not guaranteed or endorsed by the publisher.
